# Preventive Efficacy of Dried Lime (*Citrus aurantifulia*) in Common Cold Among Hajj Pilgrims: A Randomized, Double-Blinded, Placebo-Controlled Clinical Trial

**DOI:** 10.31661/gmj.v0i0.1462

**Published:** 2020-01-27

**Authors:** Mehdi Pasalar, Seyed Hamdollah Mosavat, Hossein Molavi Vardanjani, Mohsen Keshavarz, Maryam Mosaffa-Jahromi, Seyed Hossein Owji, Kamran Bagheri Lankarani

**Affiliations:** ^1^Research Center for Traditional Medicine and History of Medicine, Shiraz University of Medical Sciences, Shiraz, Iran; ^2^Essence of Parsiyan Wisdom Institute, Traditional Medicine and Medicinal Plant Incubator, Shiraz University of Medical Sciences, Shiraz, Iran; ^3^Pharmaceutical Sciences Research Center, Shiraz University of Medical Sciences, Shiraz, Iran; ^4^Health Policy Research Center, Shiraz University of Medical Sciences, Shiraz, Iran; ^5^Department of Persian Medicine, School of Persian Medicine, Tehran University of Medical Sciences, Tehran, Iran; ^6^Student Research Committee, Shiraz University of Medical Sciences, Shiraz, Iran

**Keywords:** Citrus aurantifulia, Common Cold, Coryza, Clinical Trial, Herbal Medicine

## Abstract

**Background:**

Dried lime (*Citrus aurantifulia*) is one of the herbal preparations used especially by Iranian pilgrims as a preventative agent and self-remedy for respiratory tracts symptoms in folklore medicine. Therefore, we evaluated the preventive efficacy of dried lime preparation in common cold among Iranian pilgrims.

**Materials and Methods:**

In this randomized, double-blinded, clinical trial patients in the drug group received dried lime capsules, 500 mg in a single dose per day for four weeks. In the placebo group, the patients received placebo capsules using the same method. The primary outcome measure in this trial was the severity of cold symptoms assessed by a self-administered questionnaire.

**Results:**

There were no significant differences between the two groups in terms of the trend of cold symptoms severity during the study period. However, in the second week, the severity of all the cold symptoms in the drug group was less, compared to the placebo, but at the end of the study, comparison of the two groups revealed no significant difference in any of the investigated options.

**Conclusion:**

The findings revealed that although the severity of all the cold symptoms in the drug group was less as compared to the placebo group, the dried lime capsule showed no statistically significant effect on the control of these symptoms in Iranian pilgrims.

## Introduction

Hajj is one of the most important and common religious rituals among Muslims that is held in Mecca, Saudi Arabia annually in a specific period. More than two million pilgrims from different countries participate in this ritual [1]. Overcrowding of pilgrims in a semi-closed setting at the Hajj, old age of the majority of the pilgrims, and lack of adequate health care facilities cause the pilgrims to be susceptible to communicable diseases [2]. Viral respiratory infection is one of the most common communicable diseases among Hajj pilgrims [3]. Previous studies estimated that more than 30% of Hajj pilgrims would experience respiratory symptoms during this ritual [4]. Given the high prevalence of the disease among the pilgrims and morbidity of the disease usually causing the Hajj pilgrims not to be able to do the rites of Hajj well, many pilgrims use preventative methods or self-treatment to get rid of this disease. Dried lime ( *Citrus aurantifulia*) is one of the herbal preparations that is used, especially by Iranian pilgrims, as a preventative agent and self-remedy for respiratory tracts symptoms in folklore medicine. There is also some evidence in traditional Persian medicine about the effectiveness of lime in lung diseases [5,6]. Furthermore, recent studies have demonstrated the antimicrobial effects of this plant [7,8]. Therefore, we aimed to evaluate the preventive efficacy of dried lime preparation in common cold among Iranian pilgrims in a randomized, double-blinded, placebo-controlled clinical trial during the Hajj period.

## Materials and Methods

###  Trial design 

 This was a randomized, double-arm, parallel group, double-blind clinical trial that was approved by Local Medical Ethics Committee of Shiraz University of Medical Sciences (approval ethics number: EC2798) in September 2014 and registered at Iranian Registry of Clinical Trials (IRCT20170704034897N2).

###  Participants

 The participants were included in this study based on the following criteria: men and women attending the Hajj ceremony and aged 35 to 80 years. Exclusion criteria were the presence of asthma and chronic obstructive pulmonary disease, heart diseases, heart failure, hepatic and renal failure, and history of previous chest surgery.

###  Intervention

 The enrolled patients were randomly assigned to receive dried lime capsule (n = 60) as the drug group or placebo group (n = 52). Patients in the drug group received dried lime capsules, 500 mg in a single dose per day for four weeks. In the placebo group, the patients received placebo capsules using the same method.

###  Preparation of drugs

The test drug consisted of *C. aurantifulia* and sugar. The dried fruit of *C. aurantifulia* was purchased from the green market in Shiraz (Iran), authenticated by a botanist (voucher number: 35-8547). A 500 mg of the milled powder of the dried fruit of *C. aurantifulia* and 50 milligrams of sugar were used as filler filled into a capsule, weighing 550 mg. The placebo capsule consisted of 550 mg of flour with identical size and shape to test the drug.

###  Outcome 

 The primary outcome measure was the severity of cold symptoms assessed by a self-administered questionnaire. The questionnaire evaluated the cough, rhinorrhea, fever, and body pain that ranged from 0-5 scores (without manifestation to severe symptom).

###  Randomization

 One hundred and twelve eligible patients were randomized into two parallel groups with simple block randomization method. A general practitioner visited all the patients, and the enrolled participants in the trial were assigned to the drug or placebo group, according to the randomization list.

###  Statistical Analysis

Data were analyzed using descriptive statistics. Chi-square and two independent sample T-tests were used for statistical comparison of the baseline characteristics. Outcome variables were dichotomized as “who had a common cold” vs. “who had not.” To determine the effects of the intervention on the patients’ outcome, we analyzed the data applying General Estimation Equation modeling technique. The P-value less than 0.05 was considered significant. All the data were analyzed using STATA, version 11.2 (Stata Corp. LP, TX, USA) [9].

## Results

**Figure 1:**
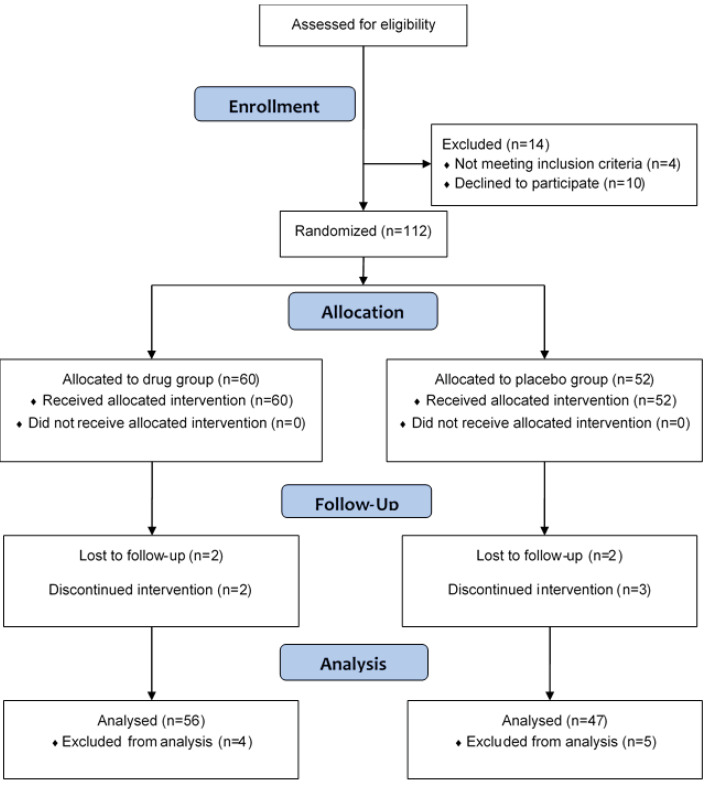
Flow diagram of study

**Figure 2:**
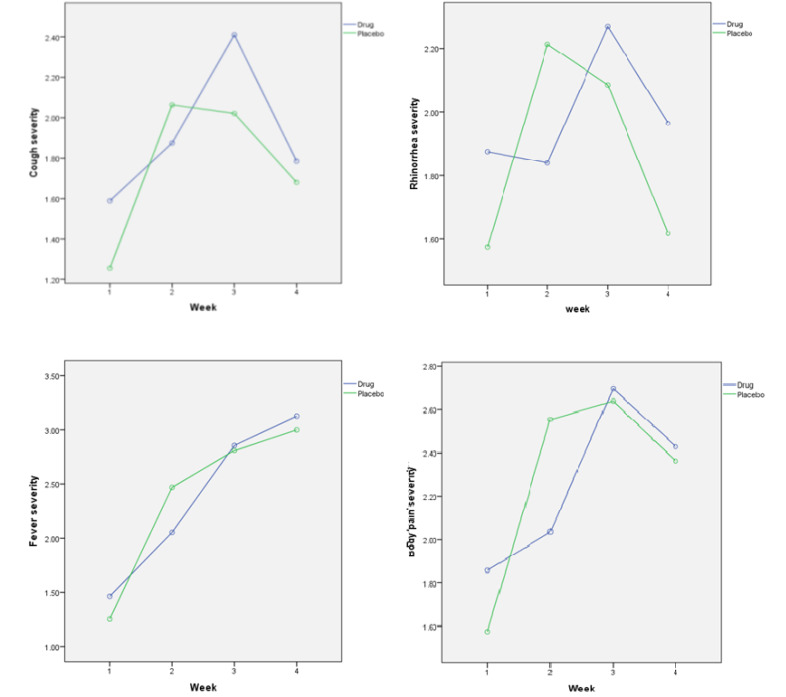
Trends of the severity of cold symptoms during the study period in both groups of the study

 One hundred twenty-six volunteers were assessed for eligibility. One hundred and twelve volunteers were divided into two groups. Sixty volunteers were assigned to the drug group and 52 to the placebo group. Figure-1 is the flowchart of the group’s distribution, recruitment, intervention, follow-up, and analysis. The mean age of the participants was 54.50±11.41 and 50.46±10.26 years in the drug and placebo groups, respectively (P=0.06). Furthermore, the male/female ratio was 35/21 and 28/19 in the drug and placebo groups, respectively (P=0.84). Moreover, the percentage of vaccinated participants was 30.4% and 36.2% in the drug and placebo groups, respectively (P=0.67). No significant differences were observed in the baseline demographic data between the two groups of the study. As shown in Figure-2, there were no significant differences between the two groups in terms of the trend of the severity of the cold symptoms during the study period. However, in the second week, the severity of all the cold symptoms in the drug group was less, compared to the placebo, but at the end of the study, comparison of the two groups revealed no significant difference in any of the investigated options. The review of the patient’s files showed that there were no reported adverse events in the study and placebo groups during the trial.

## Discussion

Based on Persian medicine resources, dried lime is an important component of respiratory system prescriptions. It can reduce the routine respiratory symptoms like rhinorrhea, cough, sore throat, myalgia, and so on. The mechanism of action is not clear enough although there are some holistic theories to shed light on the subject [10,11]. In recent literature, dried lime is a good choice of treatment in common cold due to its antimicrobial properties against gram-positive or gram-negative germs. Antifungal properties are also seen in recent studies [7,12]. This may explain its efficacy as a suitable agent against upper respiratory tract infections. The antioxidant properties of the dried lime essential oil with their multiple mechanisms mainly attributed to its terpenoid content should be kept in mind in this context, as well [13]. Viral upper respiratory tract infection is the most commonly reported disease at the Hajj [14,15]. Thus, many pilgrims try to prevent respiratory tract infections during the Hajj using herbal preparations. To the best of our knowledge, no clinical trial has evaluated the preventive efficacy of herbal or conventional medicine on respiratory diseases of Hajj pilgrims. Some studies investigated the effectiveness of face masks in preventing respiratory viral infection among Hajj pilgrims [16,17]. Perhaps, one of the reasons for lack of sufficient similar clinical studies is that pilgrims hardly agree to participate in the clinical research during their pilgrimage. Therefore, one of the strong points of this study is accomplishing a clinical trial on pilgrims during the Hajj period. Another two strong points of this study were long term treatment period (4 weeks) and the use of placebo in the design of the study. Several Chinese herbal preparations have been investigated in the past for alleviating the common cold symptoms [18-20]. These studies have shown that Chinese herbal medicines could shorten the symptomatic phase of the common cold [18]. Therefore, the result of our research is concomitant with the previous similar studies because in our study, in the second week, the severity of all the cold symptoms in the drug group was less as compared to placebo. Of course, most of the previous studies had some limitations. Lack of placebo comparator arm in the design of most of the previous studies is one of the most important limitations, and this issue was considered in this study, so placebo comparator is another strong point of this study compared to previous studies.

## Conclusion

 The findings revealed that although the severity of all the cold symptoms in the drug group was less as compared to the placebo, the dried lime capsule showed no statistically significant effect on the control of these symptoms in Iranian pilgrims.

## Acknowledgment

 The authors would like to thank the research vice chancellery of Shiraz University of Medical Sciences for funding supports (grant no.: 133). The authors thank Dr. N. Shokrpour for the language editing of this manuscript.

## Conflict of Interest

 None of the authors have a conflict of interest to declare.
